# Supplementary material for: Systematic Review and Meta-Analysis of Electromyography Potential to Discriminate Muscular or Articular Temporomandibular Disorders and Healthy Patients
Source: Healthcare (Basel). 2025 Feb 21;13(5):466. doi: 10.3390/healthcare13050466 (PMC11899047; doi:10.3390/healthcare13050466)
Supplement: Supplementary file 1 [file healthcare-13-00466-s001.zip › Table S1.pdf]

Supplementary Table S1

**Strategy search**
**PubMed**
((((("temporomandibular joint disorders"[MeSH Terms] OR ("temporomandibular"[All Fields] AND "joint"[All Fields] AND "disorders"[All Fields]) OR "temporomandibular joint disorders"[All Fields] OR "TMD"[All Fields] OR "orofacial pain"[All Fields] OR "craniomandibular disorders"[All Fields]) AND ("electromyography"[MeSH Terms] OR "electromyography"[All Fields] OR "electromyographies"[All Fields])) NOT ("case reports"[Publication Type] OR "case reports"[All Fields])) NOT ("systematic review"[Publication Type] OR "systematic reviews as topic"[MeSH Terms] OR "systematic review"[All Fields])) NOT ("meta analysis"[Publication Type] OR "meta analysis as topic"[MeSH Terms] OR "meta analysis"[All Fields])) AND (y_10[Filter])
**Scopus**
(TITLE-ABS-KEY ("temporomandibular joint disorders") AND TITLE-ABS-KEY ("electromyography" OR "EMG") AND TITLE-ABS-KEY ("orofacial pain" OR "craniomandibular disorders") AND NOT TITLE-ABS-KEY (systematic AND review AND [doctype] OR review AND [doctype] OR meta-analysis AND [doctype] OR comment AND [doctype] OR congress AND [doctype] OR editorial AND [doctype] OR case AND reports AND [doctype] OR clinical AND conference AND [doctype] OR comment AND [doctype] OR consensus AND development AND conference AND [doctype])) AND PUBYEAR > 2011 AND PUBYEAR < 2023 AND (LIMIT-TO (DOCTYPE , "ar"))
**Web of Science**
TS=((("temporomandibular joint disorders") AND ("electromyography" OR "EMG")) AND ("orofacial pain" OR "craniomandibular disorders")) AND LANGUAGE: (English OR Italian) AND DOCUMENT TYPES: (Article) Timespan=2012-2022
