# Supplementary material for: Systematic Review and Meta-Analysis of Electromyography Potential to Discriminate Muscular or Articular Temporomandibular Disorders and Healthy Patients
Source: Healthcare (Basel). 2025 Feb 21;13(5):466. doi: 10.3390/healthcare13050466 (PMC11899047; doi:10.3390/healthcare13050466)
Supplement: Supplementary file 1 [file healthcare-13-00466-s001.zip › Table S2.pdf]

Supplementary Table S2

	Item 1	Item 2	Item 3	Item 4	Item 5	Item 6	Item 7	Item 8	Item 9	Item 10	Item 11	Item 12	Item 13	Final Score
**Berni 2015**	3	3	1	1	2	1	3	3	3	3	3	N/A	1	27/36
**Chaves 2017**	2	3	3	2	2	2	3	3	3	2	3	N/A	2	30/36
**De Paiva 2022**	3	3	3	3	3	2	3	3	3	3	3	N/A	2	34/36
**Di Giacomo 2020**	3	3	3	3	3	2	3	3	3	3	3	N/A	3	35/36
**Ferreira 2014**	3	3	3	3	3	2	3	3	3	2	3	N/A	2	33/36
**Hu 2020**	2	3	3	3	1	2	2	2	3	3	3	N/A	3	30/36
**Iodetti 2014**	3	2	2	3	1	2	3	3	3	2	3	N/A	3	30/36
**Iwasaki 2015**	2	3	2	2	1	2	2	2	2	1	3	N/A	3	25/36
**Iwasaki 2017**	2	3	2	2	2	2	2	2	2	2	3	N/A	3	27/36
**Mapelli 2016**	3	3	3	2	3	2	3	3	3	2	3	N/A	2	32/36
**Pires 2018**	2	3	3	3	2	2	2	2	3	2	3	N/A	3	30/36
**Politti 2016**	3	3	3	3	2	2	3	3	3	2	3	N/A	1	31/36
**Ries 2016**	2	3	2	3	2	2	3	3	2	0	3	N/A	3	28/36
**Rodrigues 2015**	3	3	3	3	2	2	3	3	3	2	3	N/A	2	32/36
**Santana Mora 2014**	3	3	2	3	3	2	3	3	3	2	3	N/A	3	33/36
**Serrano 2017**	3	3	3	3	3	2	3	3	3	3	3	N/A	3	35/36
**Valentino 2021**	3	3	3	2	3	2	3	3	2	2	3	N/A	1	30/36
**Xu 2017**	3	3	3	2	3	2	3	3	3	3	3	N/A	2	33/36
