# Supplementary figures and images for: Systematic Review and Meta-Analysis of Electromyography Potential to Discriminate Muscular or Articular Temporomandibular Disorders and Healthy Patients
Source: Healthcare (Basel). 2025 Feb 21;13(5):466. doi: 10.3390/healthcare13050466 (PMC11899047; doi:10.3390/healthcare13050466)

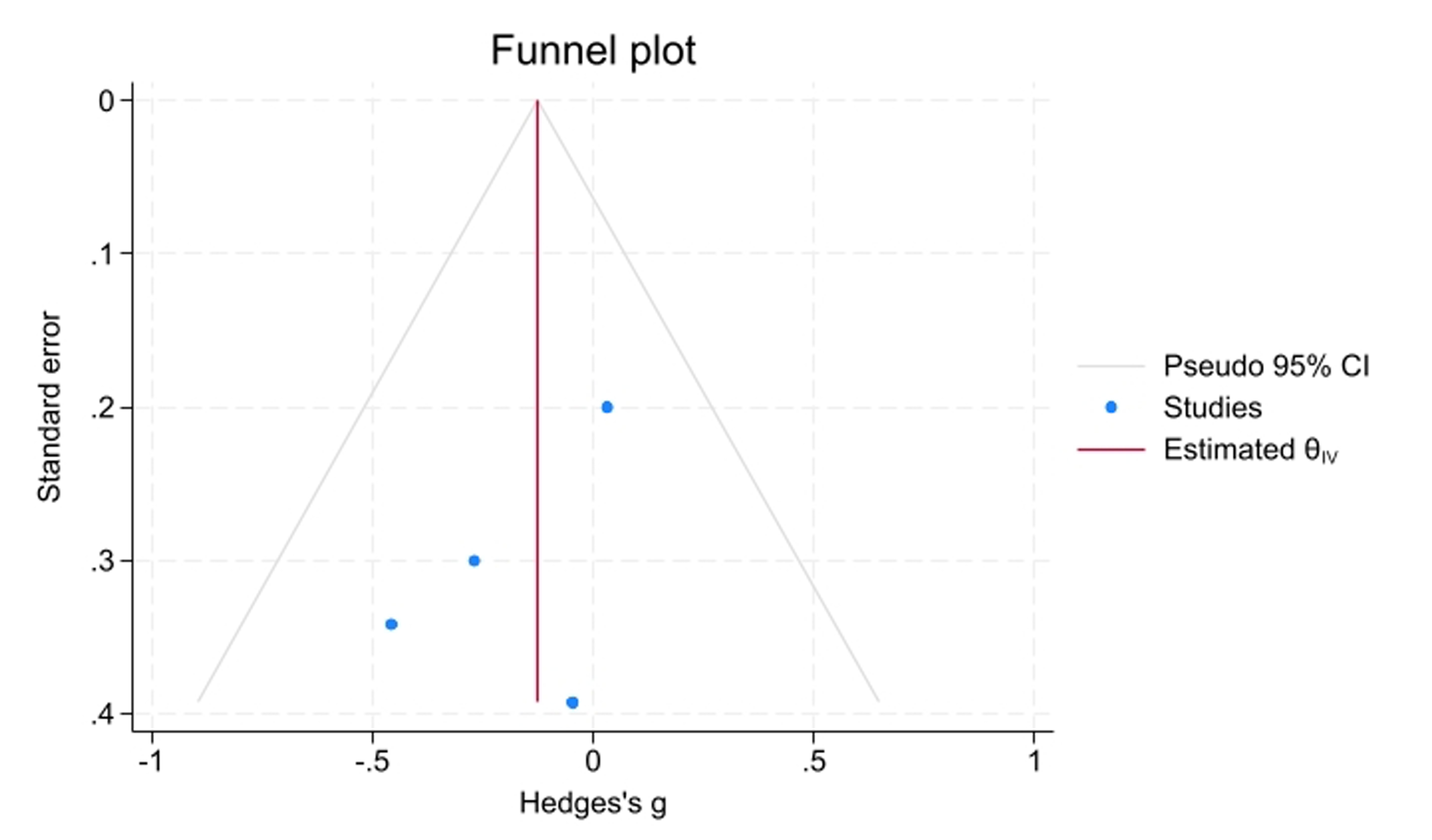

Supplement: Supplementary file 1 [file healthcare-13-00466-s001.zip › Figure S1.jpg]

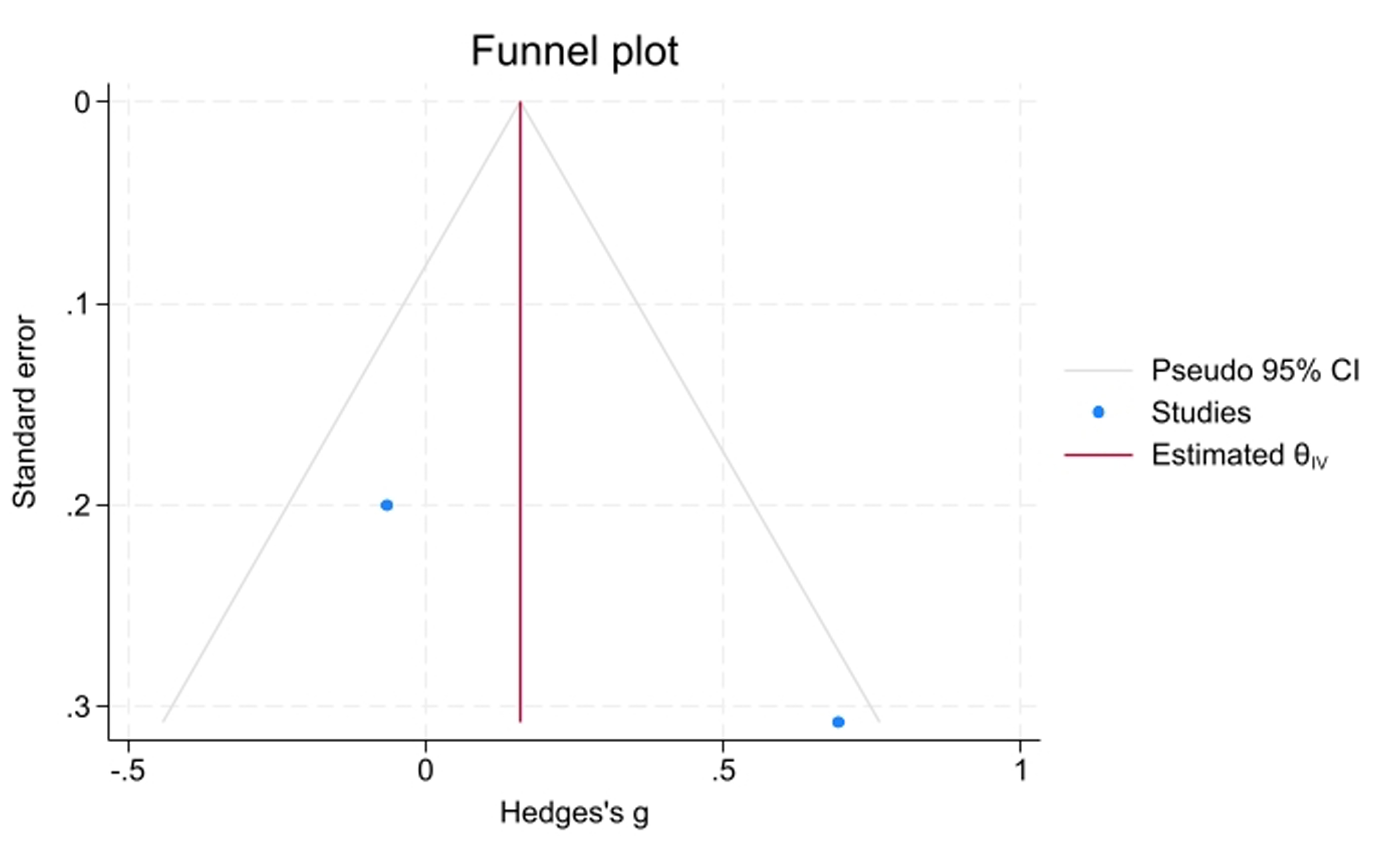

Supplement: Supplementary file 1 [file healthcare-13-00466-s001.zip › Figure S2.jpg]

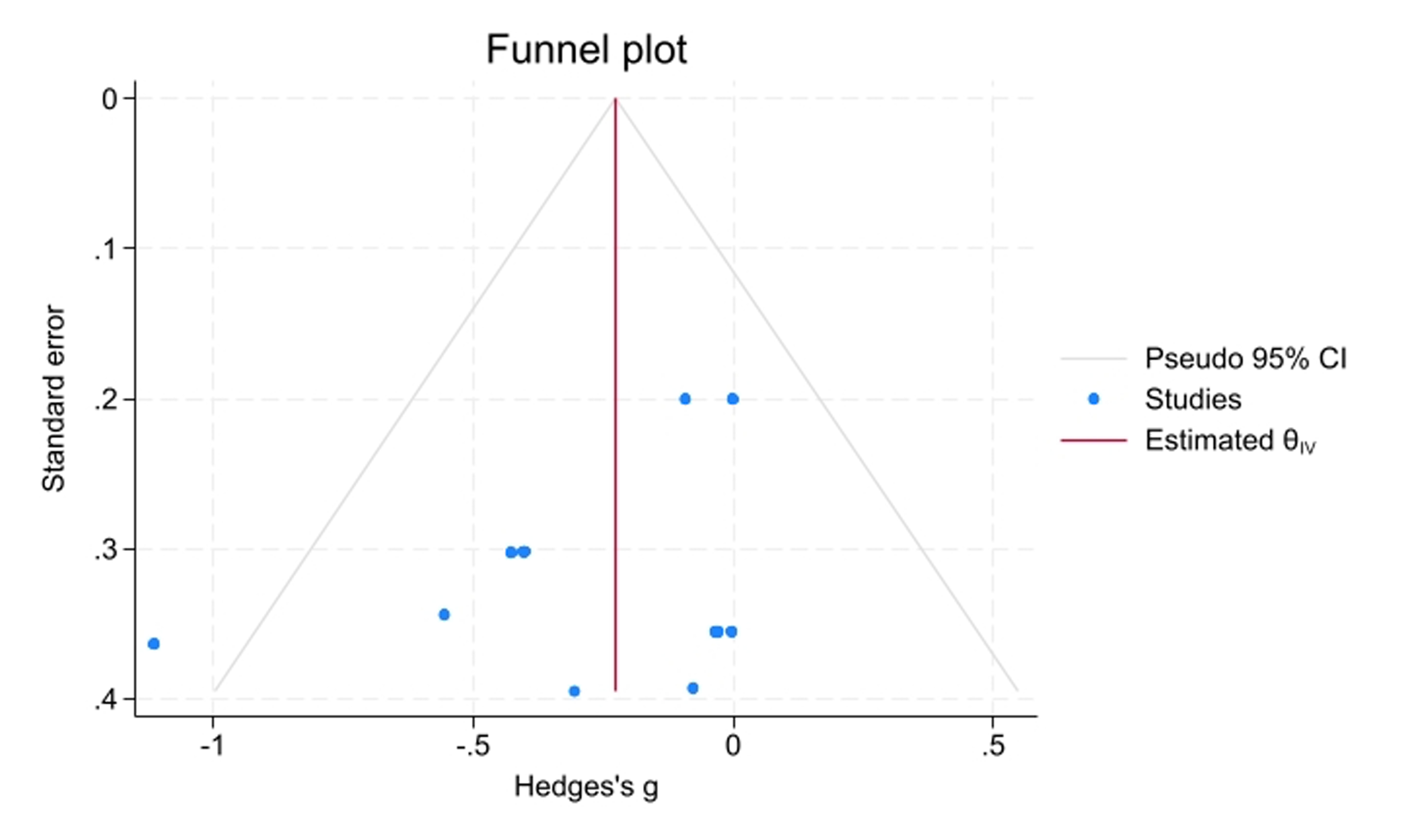

Supplement: Supplementary file 1 [file healthcare-13-00466-s001.zip › Figure S3.jpg]

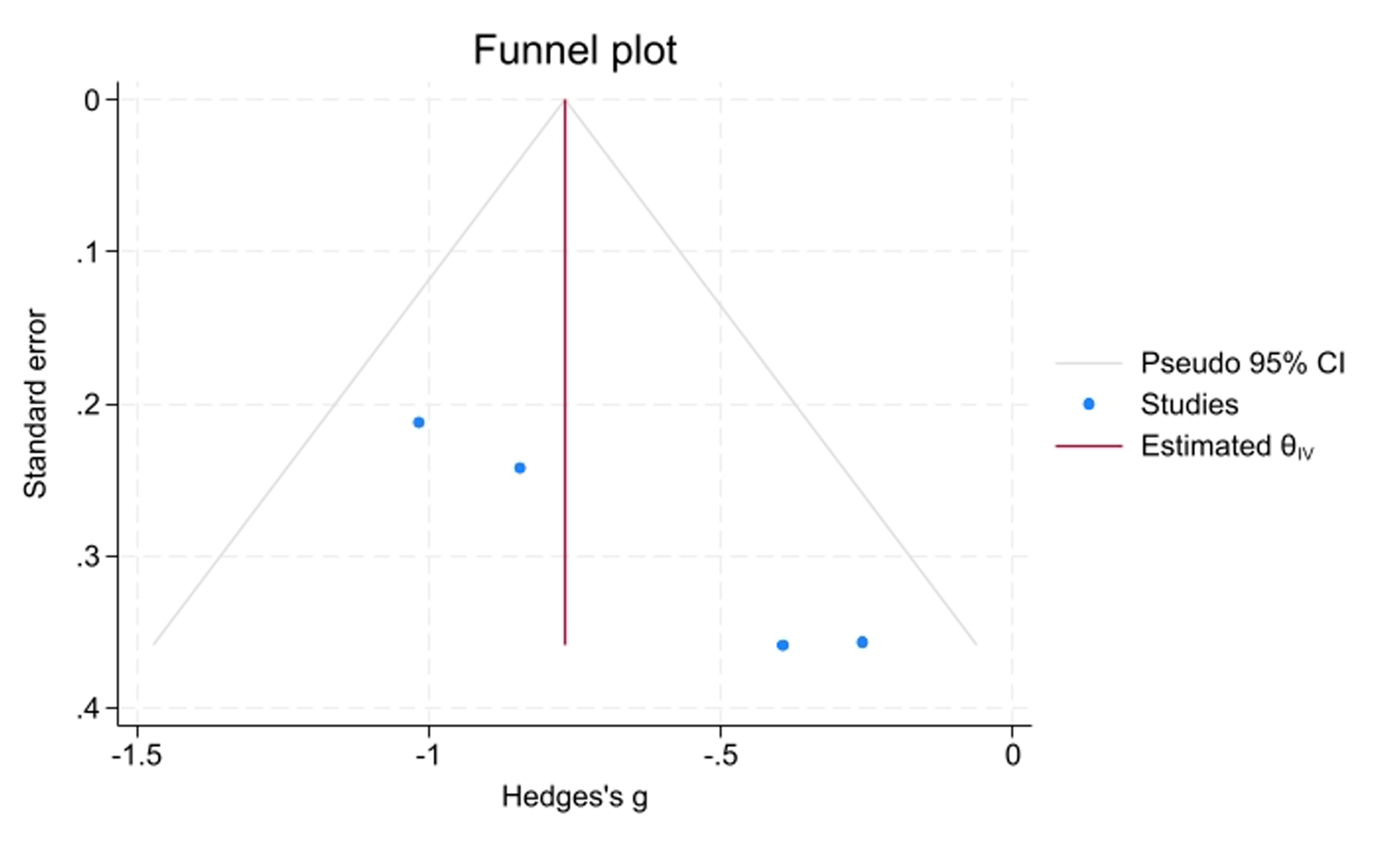

Supplement: Supplementary file 1 [file healthcare-13-00466-s001.zip › Figure S4.jpg]
